# Objective assessment of impulse control disorder in patients with Parkinson’s disease using a low-cost LEGO-like EEG headset: a feasibility study

**DOI:** 10.1186/s12984-021-00897-1

**Published:** 2021-07-02

**Authors:** Yuan-Pin Lin, Hsing-Yi Liang, Yueh-Sheng Chen, Cheng-Hsien Lu, Yih-Ru Wu, Yung-Yee Chang, Wei-Che Lin

**Affiliations:** 1 Institute of Medical Science and Technology, National Sun Yat-sen University, Kaohsiung, Taiwan; 2 Department of Electrical Engineering, National Sun Yat-sen University, Kaohsiung, Taiwan; 3 Department of Diagnostic Radiology, Kaohsiung Chang Gung Memorial Hospital, and Chang Gung University College of Medicine, Kaohsiung, Taiwan; 4 Department of Neurology, Kaohsiung Chang Gung Memorial Hospital, and Chang Gung University College of Medicine, Kaohsiung, Taiwan; 5 Department of Neurology, Linkou Chang Gung Memorial Hospital, Chang Gung University College of Medicine, Taoyuan, Taiwan; 6 Department of Diagnostic Radiology, Kaohsiung Chang Gung Memorial Hospital, No. 123, Dapi Road, Niaosong District, Kaohsiung City, 833 Taiwan

**Keywords:** Parkinson’s disease, Impulse control disorders, Electroencephalogram, Event-related potential, LEGO-like headset

## Abstract

**Background:**

Patients with Parkinson’s disease (PD) can develop impulse control disorders (ICDs) while undergoing a pharmacological treatment for motor control dysfunctions with a dopamine agonist (DA). Conventional clinical interviews or questionnaires can be biased and may not accurately diagnose at the early stage. A wearable electroencephalogram (EEG)-sensing headset paired with an examination procedure can be a potential user-friendly method to explore ICD-related signatures that can detect its early signs and progression by reflecting brain activity.

**Methods:**

A stereotypical Go/NoGo test that targets impulse inhibition was performed on 59 individuals, including healthy controls, patients with PD, and patients with PD diagnosed by ICDs. We conducted two Go/NoGo sessions before and after the DA-pharmacological treatment for the PD and ICD groups. A low-cost LEGO-like EEG headset was used to record concurrent EEG signals. Then, we used the event-related potential (ERP) analytical framework to explore ICD-related EEG abnormalities after DA treatment.

**Results:**

After the DA treatment, only the ICD-diagnosed PD patients made more behavioral errors and tended to exhibit the deterioration for the NoGo N2 and P3 peak amplitudes at fronto-central electrodes in contrast to the HC and PD groups. Particularly, the extent of the diminished NoGo-N2 amplitude was prone to be modulated by the ICD scores at Fz with marginal statistical significance (*r* = − 0.34, *p* = 0.07).

**Conclusions:**

The low-cost LEGO-like EEG headset successfully captured ERP waveforms and objectively assessed ICD in patients with PD undergoing DA treatment. This objective neuro-evidence could provide complementary information to conventional clinical scales used to diagnose ICD adverse effects.

## Background

Parkinson’s disease (PD) is a progressive neurodegenerative disorder characterized by the loss of midbrain dopaminergic neurons and the subsequent depletion of dopamine levels in the basal ganglia [1]. Patients with PD manifest the hallmarks of motor control dysfunction, i.e., tremor, bradykinesia, and rigidity. Moreover, the disorder is frequently accompanied by a cognitive decline [2, 3] in many aspects, including inhibitory control, attention shift, reward learning, and working memory; particularly, the main pharmacological treatment for the motor symptoms, e.g., dopamine agonists (DA), may trigger impulse control disorders (ICDs) as an adverse effect [4–8]. ICDs refer to the inability to inhibit predominant behaviors, thereby leading to several compulsive or pathological behavioral changes regarding gambling, shopping, eating, and sexuality [4]. The inhibitory control is the capability of selecting the most appropriate response while suppressing competing counterparts in ever-changing circumstances; its integrity is critical for controlling behavior at all levels [4, 8]. Therefore, assessing, monitoring, and ideally avoiding ICD in patients with PD has become increasingly important [4, 7–9].

Noteworthily, the ICD adverse effect can be mitigated and even terminated by reducing DA dose or switching to another dopamine replacement therapy [4]. Presently, assessing ICD mainly relies on subjective clinical judgment associated with interview outcomes of patients with PD, as well as their self-reported questionnaire scores. However, behavioral scales are potentially biased and may be inaccurate at early stages. Recent advances in neuroimaging facilitate the exploration of impulse control-relevant neural networks and their interaction with psychopharmacological interventions. Several neuroimaging techniques including near-infrared spectroscopy [10], functional magnetic resonance imaging [11, 12], and electroencephalogram (EEG) [13, 14] have demonstrated the feasibility of associating neurological evidence with inhibitory control. Therefore, it is possible to find brain-markers that objectively characterize cognitive decline in neurodegenerative diseases [15]; for example, impulse control integrity in patients with PD during chronic DA treatment.

Among the available neuroimaging techniques, EEG measures the electrical brain activity with a high temporal resolution of milliseconds, which captures the onset of cognitive states and their rapid transitions. Moreover, wearable EEG-recording technology has recently made impressive progress. Unlike the bulky gel electrode-headset of laboratories, wearable technology allows the recording of brain activity using dry/saline electrodes, wireless transmission, and a minimized amplifier [16–18]. Furthermore, the easy-to-setup wearability makes the EEG measurement more user-friendly, less headset-calibrated, and considerably promotes realistic EEG applications in daily life [19–21].

Event-related potential (ERP) is a well-established signaling marker related both to the qualitative and quantitative assessment of cognitive processes. During EEG recording, the individual undergoes a task-specific experimental protocol to study a cognitive capacity of interest for sequential ERP analysis. The alteration of ERP waveforms has been previously linked to the integrity of the targeted cognitive function. For example, the oddball paradigm is a classic task that engages the selective attention network. When the brain perceives a deviant target stimulus, the P3 component (a positive peak around 300–500 ms following stimulus onset) dominantly appears at the midline scalp electrodes [22, 23]. Either an attenuated or absent P3 component could implicate alterations or even deficits in attention shifting [3, 24]. The Go/NoGo task is another common task that investigates both cognitive and motoric inhibition [25, 26]. While frequent Go trials are characterized by an as-fast-as-possible behavioral response, the rare NoGo trials imply a withholding of the prepotent response, i.e., inhibition control. The successful NoGo inhibition normally leads to clear N2 (negativity around 200–300 ms) and P3 signals at fronto-central regions as compared with Go trials [13, 25, 26]. Consistent with the aforementioned, diminished amplitudes of N2 and P3 components are associated with dysfunction in inhibition control in individuals with attention-deficit/hyperactivity disorder [27], internet addiction [28], and PD [14]. Accordingly, the ERP signature during an actively engaged cognitive task is capable of examining deficits in the targeted cognitive function regarding physiological, psychological, and psychiatric disorders.

Motivated by the ERP assessment of cognitive capacity and its applicability to Go/NoGo task-engaged inhibition control, we attempted the application of the ERP signaling strategy to reveal ICD neural signatures in patients with PD who undergo DA pharmacological interventions. We hypothesized that patients with PD and ICD comorbidity would exhibit diminished amplitudes of the N2 and P3 complex after DA treatment as compared with typical patients with PD. This amplitude degradation is potentially related to ICD severity, that is, ICD symptom severity being positively associated with diminished amplitude. A further objective was to approach the above issue using a customized, cost-efficient EEG amplifier [29] and electrode-holder assembly [30] (namely, a LEGO-like EEG headset). Using a non-medical-grade EEG-sensing platform is a harsh, yet, realistic setup, closer to a practical wearable device for ubiquitous ICD monitoring. While most studies focused either on the EEG/ERP distinction between patients with PD and healthy participants [14, 31, 32] or between different PD stages [33–35], only a few studies devoted to the neural assessment of ICD in patients with PD. A recent study [36] focused on the exploration of the spatio-spectral dynamical patterns produced by high-density EEG recordings (i.e., 128 channels) and pinpointed the underlying neural mechanisms associated with inhibitory control dysfunction in patients with PD and ICD comorbidity compared with patients with PD alone. Conversely, this work attempted both to determine whether ERP signatures can objectively reflect the severity of the DA-triggered ICD adverse effect in patients with PD and practically contribute to the validation of using a customized, cost-efficient EEG-sensing setup as opposed to an expensive laboratory-oriented or medical-grade product. Successful results will elucidate how an EEG-based wearable device can routinely monitor ICD symptoms in each patient with PD at home, and how it may, thereby, guide clinical practice to an optimal DA dose management or pharmacological plan-establishment while treating motor symptoms.

## Methods

### Participants

We recruited 59 participants who were divided into three groups of 23 patients with PD (PD group: 16 males, 7 females), 10 patients with PD and ICD comorbidity (ICD group: 8 males, 2 females), and 26 healthy controls (HC group: 13 males and 13 females). All patients were interviewed by experienced neurologists and diagnosed with idiopathic PD according to the United Kingdom Brain Bank criteria [37]. We excluded patients with a history of other neurologic and/or psychiatric illnesses and use of psychotropic medications. The Unified Parkinson’s Disease Rating Scale (UPDRS) [38], the modified Hoehn and Yahr (H&Y) Staging Scale [39], and the Schwab and England (S&E) Activities of Daily Living Scale [40] were employed in the evaluation of both disease severity and functional status for multiple PD aspects. Moreover, each participant completed the Questionnaire for Impulsive-Compulsive Disorders in the Parkinson’s Disease Rating Scale (QUIP-RS) [41], a valid and reliable rating scale for ICD, useful in monitoring its severity over time. ICD diagnoses were further confirmed via a clinical interview per the QUIP-RS score.

Data from seven participants (HC: 3, PD: 3, ICD: 1) were discarded (see subsection ‘Signal preprocessing and analysis’ for details). The demographic and clinical characteristics for each group’s remaining participants are listed in Table 1. Regarding the critical characteristics of PD severity, the UPDRS, H&Y, and S&E scores were statistically comparable with the PD and ICD groups (*p* > 0.39); however, the QUIP-RS score was significantly higher (*p* < 0.01) in the ICD than in the PD group. The participants’ self-reported QUIP-RS scores were allowed the exploration of the ERP signatures’ associations.

**Table 1:** The demographic and clinical characteristics for each group

	HC	PD	ICD	*p* value
*Demographic*				
N	23	20	9	
Sex	11 M/12 F	13 M/7 F	7 M/2 F	
Age	59.26 ± 6.85	65.85 ± 9.11	63.22 ± 7.74	0.05
Education (year)	14.52 ± 3.38	11.85 ± 3.51	12.44 ± 5.43	0.05
Disease duration (year)		6.55 ± 3.85	11.22 ± 5.80	0.04
*Clinical characteristics*				
UPDRS I		3.15 ± 2.01	2.78 ± 1.48	0.75
UPDRS II		9.05 ± 5.18	10.00 ± 5.79	0.69
UPDRS III		21.30 ± 13.10	17.33 ± 6.28	0.59
UPDRS total		33.50 ± 18.19	30.11 ± 12.53	0.74
Modified H&Y		1.70 ± 0.88	1.28 ± 0.26	0.39
S&E		78.05 ± 27.31	85.56 ± 7.26	0.67
Onset site (right/left/symmetric)		5/8/7	3/5/1	
QUIP-RS		0.45 ± 1.15	16.00 ± 12.32	< 0.01

*H&Y* Hoehn and Yahr Staging Scale, *QUIP-RS* Questionnaire for Impulsive-Compulsive Disorders in Parkinson’s Disease Rating Scale, *S&E* Schwab and England Activities of Daily Living Scale, *UPDRS* Unified Parkinson’s Disease Rating Scale (Part I/II/III/total)

Values are given as mean ± standard deviation. For statistical values, Age and Education (year) between HC, PD, and ICD groups were assessed by Kruskal–Wallis nonparametric one-way analysis of variance (Age: HC vs. PD = 0.04, HC vs. ICD and PD vs. ICD > 0.45, Education (year): HC vs. PD = 0.04, HC vs. ICD and PD vs. ICD > 0.59). Disease duration (year), UPDRS, H&Y, S&E, QUIP-RS between PD and ICD groups were assessed by Wilcoxon rank-sum test

The clinical assessment of participants with PD/ICD and their data collection took place in the Kaohsiung Chang Gung Memorial Hospital (CGMH). Both the PD and ICD groups performed the first Go/NoGo session at least 12 h after the dose of dopaminergic medication (off state). The study protocol was approved by the CGMH ethics committee. Each participant was fully informed of the research purpose and provided written consent before the experiment. No participants have previously experienced the employed experimental task. Neither the DA dose nor the treatment were altered for any patient with PD per their EEG analytical outcome.

### Go/NoGo experiment and EEG signal collection

**Fig. 1:**
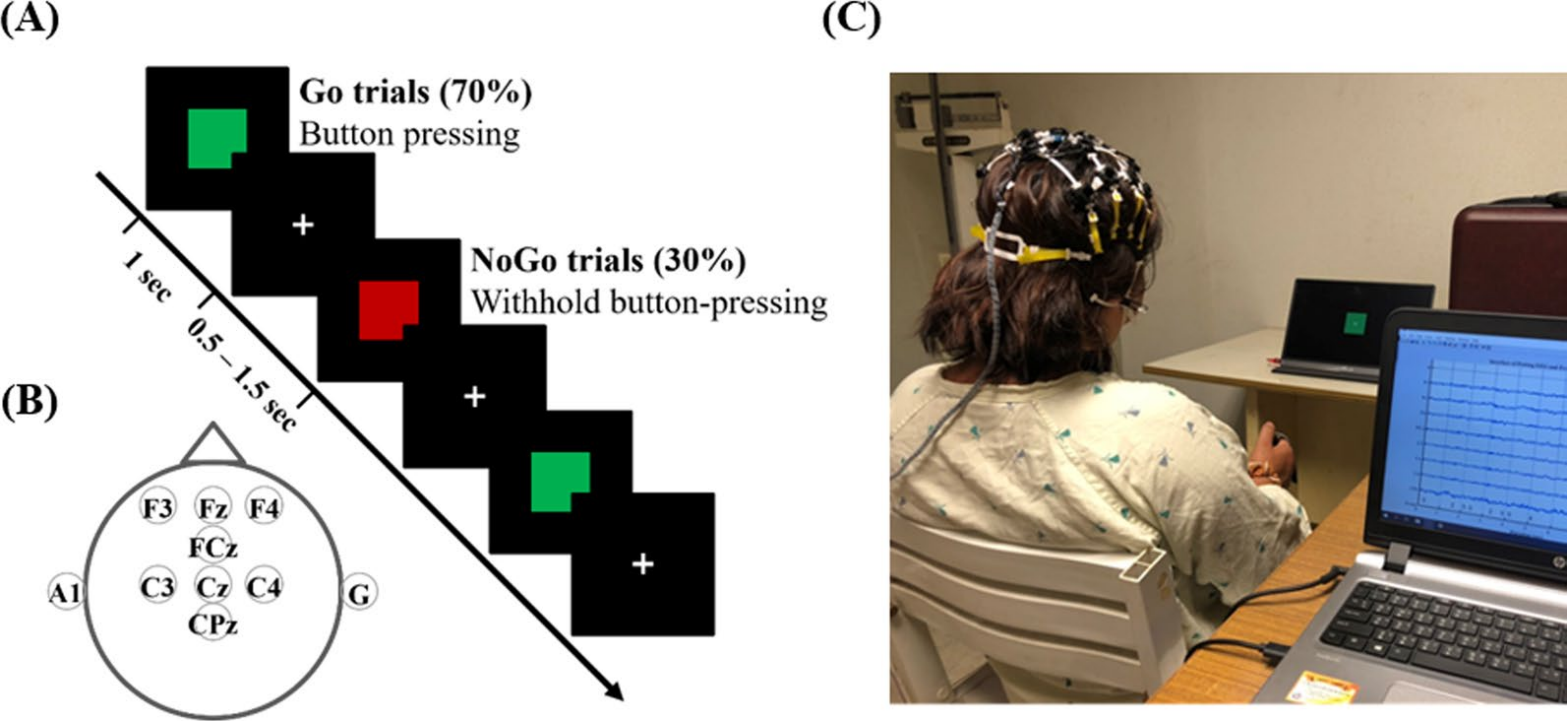
Experiment protocol and EEG recording setup. **A** The designed two-target visual Go/NoGo task, **B** the 8-channel EEG montage, and **C** a snapshot of an EEG experiment with the assembled LEGO-like headset

A two-target visual Go/NoGo task was selected to elicit cognitive and motoric inhibition during the EEG recording. The participant had to press a handheld button as quickly and accurately as possible upon the frequent presentation (70%) of a green square (i.e., the Go trials, 8.5 cm × 8.5 cm) at the center of a 16" monitor, but refrain from pressing the button upon the rare presentation (30%) of a red square (i.e., the NoGo trial). The protocol is shown in Fig. 1A. Each participant underwent the Go/NoGo task in two sessions with a ~ 1 h inter-session rest. The PD and ICD groups were administered DA medication immediately after completing the 1st session. Each session was constituted by three 80-trial blocks, lasting about 30 min. The inter-trial jitter was set between 0.5 and 1.5 s. Each session collected a total of 168 and 72 Go and NoGo trials, respectively, per participant.

We used a lab-customized, cost-efficient, portable 8-channel EEG amplifier [29] wired to a LEGO-like electrode-holder assembly [30]. The amplifier sampled the EEG signals at 250 Hz and in a bandwidth between 0.6 and 56.5 Hz. Each set of the LEGO headset (i.e., sensor positioning ring, inter-ring bridge, and bridge shield) had an 8-channel electrode-holder grid assembled and attached to position the EEG electrodes over the locations of F3, Fz, F4, FCz, C3, Cz, C4, and CPz (see Fig. 1B), mainly covering the fronto-central region relevant to the inhibition processing of the N2 and P3 components [13, 25, 26]. The assembled LEGO headset accommodated the dry electrodes (Cognionics Inc., San Diego, CA) for the measurement with respect to the left and right earlobes as ground and reference sites, respectively. Regarding the integrity of the customized EEG recording infrastructure, the portable amplifier is capable of recording ERP P3 waveforms in an auditory oddball paradigm using a hyperscanning setup for three participants with a 10-day reproducibility test [29]. Its integration to the LEGO headset has also been verified both by a steady-state visual-evoked potential (SSVEP) task [30], and the same oddball task with still and walking participants [30, 42]. Figure 1C presents the experimental setup for the Go/NoGo task and the EEG recording.

### Signal preprocessing and analysis

We adopted the following procedures to pre-process the collected EEG signals and extract N2 and P3 peak amplitudes corresponding to the Go and NoGo trials per EEG session. First, the raw EEG signals were band-pass filtered into a bandwidth of 1–30 Hz. Then, the filtered EEG signals were segmented into epochs ranging between − 200 and 1000 ms, per the visual target onset, and corrected upon their pre-stimulus baseline. The artifactual epochs with a statistical kurtosis value exceeding a threshold of 4 were rejected, followed by signal quality-ensuring via visual inspection. Moreover, the epochs corresponding to erroneous behavioral responses (standard error (SE): no button-pressing for Go trials, commission error (CE): button-pressing for NoGo trials) were discarded. Therefore, there were seven participants (HC: 3, PD: 3, ICD: 1) whose retained epochs (less than 80%) were discarded due to either technical or personal issues. The remaining 52 participants retained ~ 91% epochs on average for sequential analysis. Particularly, there were no significant differences (*p* > 0.25) in the number of the remaining NoGo trials between the three groups (63–67 trials on average) for both sessions.

We employed an independent component analysis (ICA) to remove eye movement artifacts that commonly accompany a visual task. The remaining epochs were incorporated into an extended infomax ICA algorithm to parse the 8-channel signals into independent components (ICs), of which, those with pronounced characteristics of eye movement in terms of activation waveform and spectral profile were identified and removed [43]. The remaining ICs were then back-projected to the channel-space, returning ocular artifact-suppressed EEG epochs.

Before calculating the N2 and P3 peak amplitudes, z-score standardization was applied to each EEG epoch (i.e., subtracting the mean and dividing by the standard deviation of its baseline) prior to deriving the average ERP profile of Go and NoGo conditions in each session. Then, the N2 and P3 peak amplitudes were defined within the time intervals of 200–500 ms (i.e., a maximal negative deflection in amplitude) and 400–700 ms (i.e., a maximal positive amplitude), respectively. These time intervals were selected while considering that N2 and P3 could differ between individuals and groups [14]. It is noteworthy that this work quantified the N2 and P3 signatures by peak amplitude instead of mean amplitude. The wide time intervals allowed the pinpointing of suitable peak candidates. Hereafter, the resultant N2 and P3 peak amplitudes were used to relate to the impulse control capability and explore intergroup differences both with and without pharmacological intervention. Because of the imbalanced group samples, the statistical significance of the between-session differences pertaining to behavioral outcomes was assessed by the paired sample t-test and the Wilcoxon signed-rank test for the HC/PD groups and the ICD group, respectively. Regarding the ERP outcomes, a permutation test procedure was adopted to assess the statistical significance of the between-session difference, either within each group or between groups over different channel locations, while considering the control of family-wise error rate [44, 45]. Namely, the permutation approach iteratively shuffled the N2/P3 signatures 20,000 times among the recruited group participants. Its statistical assessment was conducted by comparing the observed test statistic value against a distribution from permuting the observed values under the null hypothesis.

The EEG analytical procedures and visualization plots were performed using the open-source EEGLab toolbox/scripts [46] and in-house MATLAB scripts (The Mathworks, Inc., Natick, MA, USA).

## Results

**Fig. 2:**
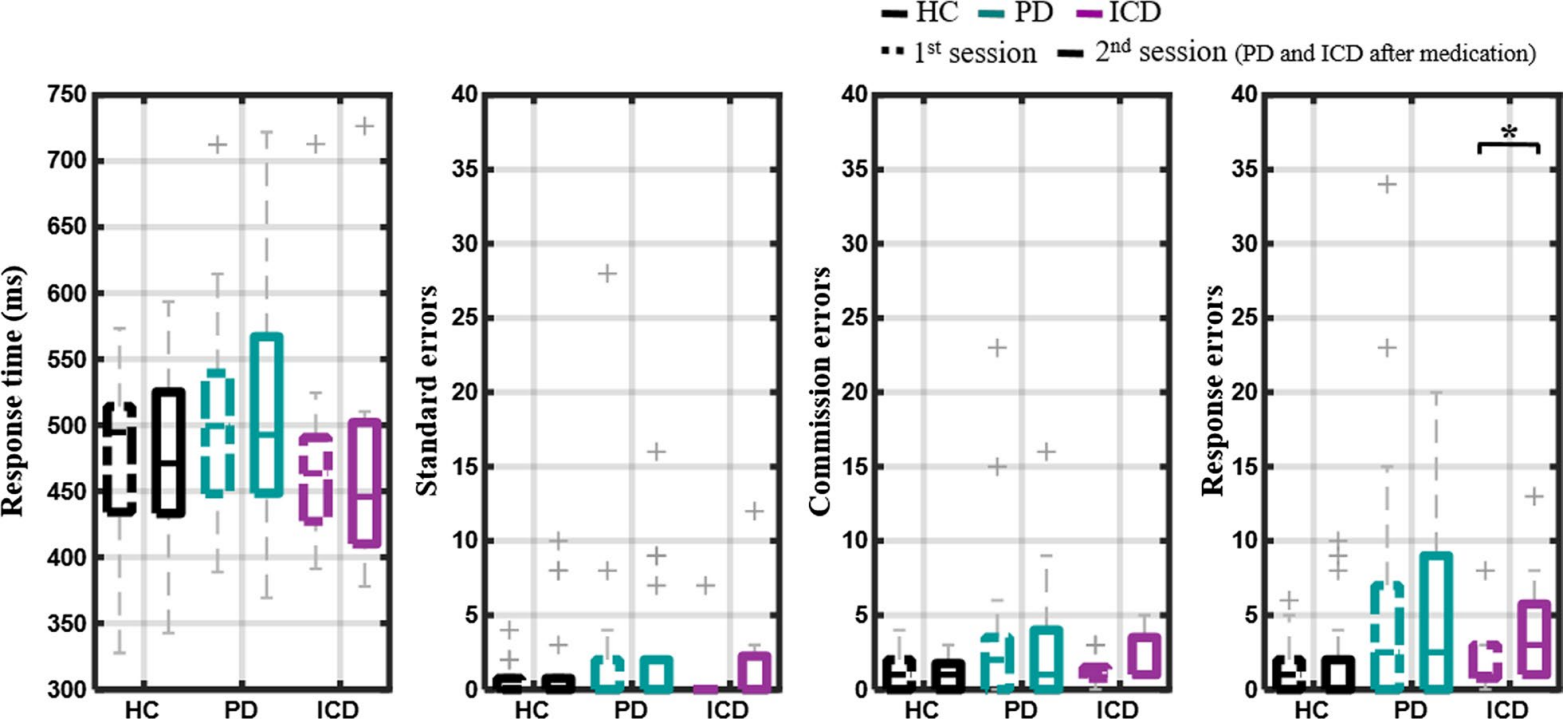
Behavioral results of the Go/NoGo task, including response time (ms) for Go trials, standard errors for Go trials, commission errors for NoGo trials, and all response errors

Figure 2 shows the behavioral outcomes of the button-pressing task, including the Go trials’ response time (RT) and standard errors, the NoGo trials’ commission errors, and all response errors to Go (SE) and NoGo (CE) trials. The ICD group was prone to faster responses to Go targets relative to the HC and PD groups, particularly on the 2nd session following their DA medication administration. There was a mean RT reduction of about 9 ms for the ICD group (1st session: 483.9 ± 93.8 ms, 2nd session: 474.5 ± 104.7 ms) with respect to an 8-ms increase for the PD group and a 4-ms reduction for the HC group; however, these RT differences were not statistically significant (*p* > 0.5). Along with such an RT outcome, the average errors in the ICD group tended to be higher for the 2nd sessions (SE: 2.11 ± 3.89; CE: 2.11 ± 1.76) compared with the 1st session (SE: 0.78 ± 2.33; CE: 1.22 ± 1.09); however, it did not statistically differ (*p* > 0.13). Both the SE and CE were relatively comparable across sessions in the PD and HC groups (SE 1st/2nd: 2.50 ± 6.35/2.35 ± 4.39; CE: 3.45 ± 5.72/2.85 ± 3.99; *p* > 0.29; SE: 0.48 ± 0.99/1.35 ± 2.99; CE: 1.04 ± 1.19/0.91 ± 0.90; *p* > 0.13, respectively). While considering SE and CE together, only the ICD group made more behavioral errors in the 2nd session than in the 1st session (2nd vs. 1st: 4.2 ± 4.1 vs. 2.0 ± 2.5; *p* = 0.03). The other two groups made comparable errors (*p* > 0.2) between the two sessions.

**Fig. 3:**
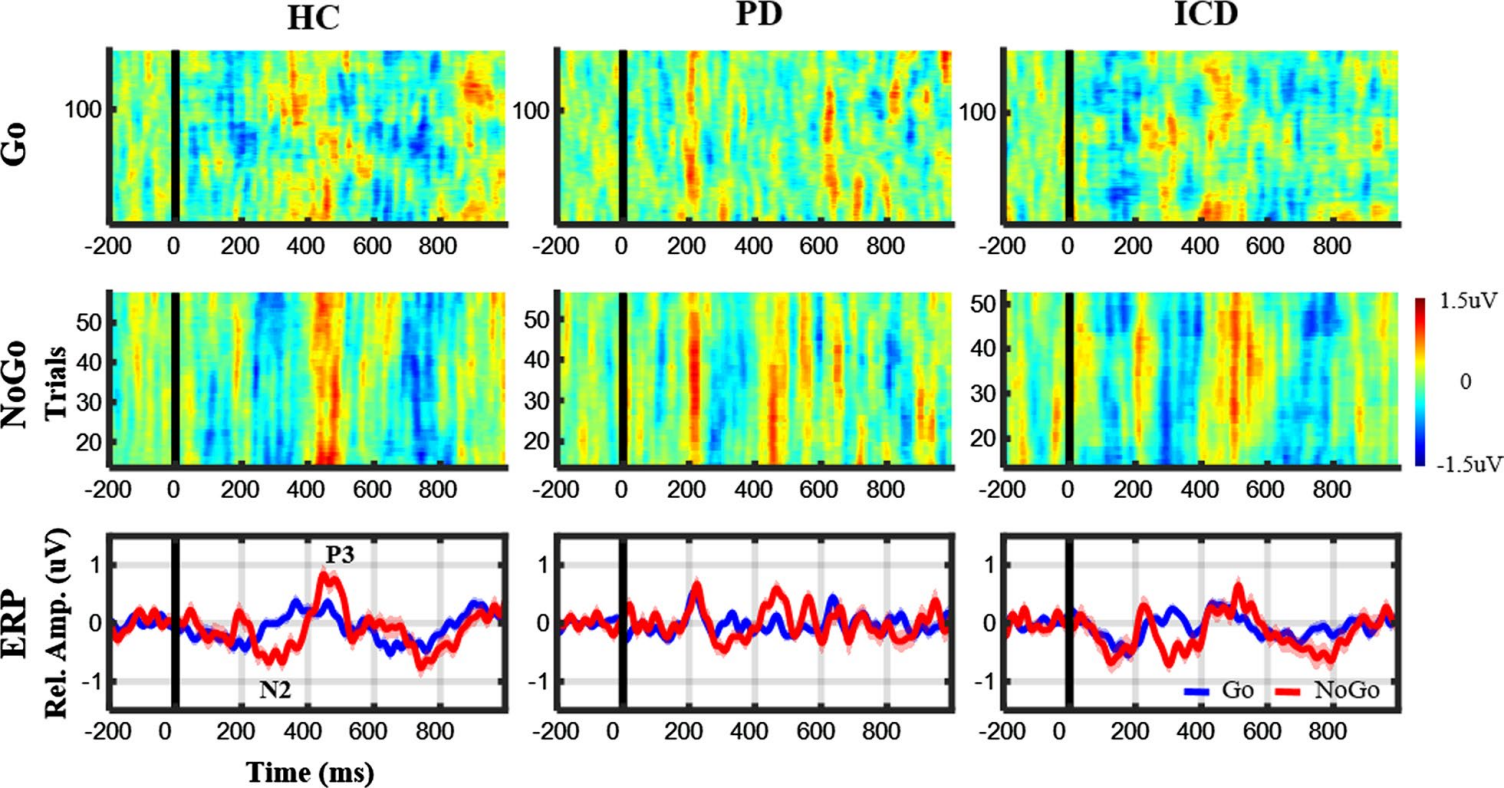
ERP images and profiles at Cz from representative subjects of HC, PD, and ICD groups. The NoGo trial (red trace) corresponds to N2 and P3 peaks around 200–400 ms and 400–600 ms, respectively, concerning the Go trials (blue trace)

Figure 3 compares the ERP profiles of the Go and NoGo trials at the Cz electrode in representative participants from the HC, PD, and ICD groups. Generally, ERP images (first two rows) show how NoGo trials present an N2 peak (blue strap) around 200–400 ms and a P3 peak (red strap) around 400–600 ms. The N2 and P3 signatures were relatively consistent across trials as compared with the Go counterpart. Note that the ERP images were displayed after smoothing 10 neighboring trials (by default in EEGLab [46]), which were intended solely for visualization purposes. After applying the synchronizing averaging to all available trials, the ERP profile (last row) exhibited N2 and P3 peaks exclusively for the NoGo condition. The Go-NoGo comparison empirically demonstrated the validity of the Go/NoGo protocol for eliciting impulse inhibition and the corresponding ERP signatures of interest.

**Fig. 4:**
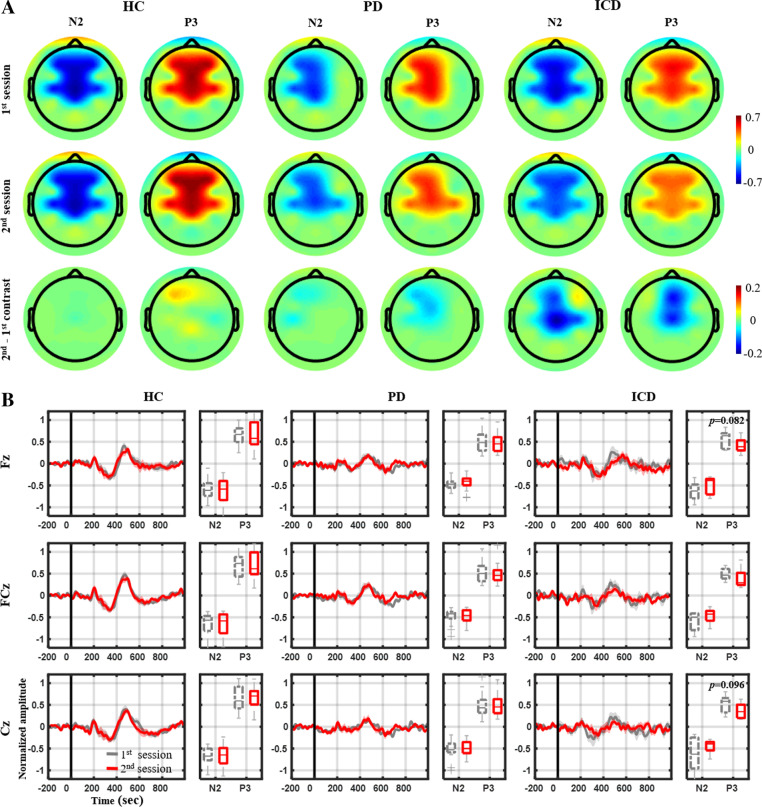
Comparative NoGo N2 and P3 signatures between the 1st and 2nd sessions and their contrast. Only the PD and ICD groups underwent DA treatment right after the 1st session. **A** Topographic mapping of peak amplitudes over the adopted 8-channel montage. The color was normalized according to the amplitudes across groups. **B** ERP profiles and peak amplitude distributions

Figure 4 further presents the topographic mapping of NoGo N2 and P3 peak amplitudes and their between-session contrast at the representative electrodes for the HC, PD, and ICD groups. The HC group exhibited relatively stronger N2 and P3 amplitudes for both sessions as compared with the other groups. The peak distributions were maximally located at the fronto-central midline electrodes (i.e., Fz, FCz, and Cz), while the P3 distribution also expanded laterally towards F3 and F4 (as shown in Fig. 4A). Furthermore, while both PD and ICD groups were administered their DA therapy after the 1st session, only the ICD group developed noticeable deterioration in N2 and P3 peak amplitudes in the 2nd session over the midline electrodes. As shown in Fig. 4B, the ICD group’s between-session P3 amplitude difference reached marginal statistical significance at Fz and Cz (*p* < 0.096), whereas the N2 contrast simply returned a tendency in decline (0.166 < *p* < 0.198). Conversely, the PD group’s behavior was comparable with the HC group, barely yielding distinguishable between-session N2 and P3 contrasts (0.384 < *p* < 0.948). Along with the amplitude outcome, Table 2 summarizes the latencies of N2 and P3 peaks for each group. Generally, the peak latency did not substantially differ (*p* > 0.18) across the two sessions for most cases in terms of the ERP components and electrode locations. Only the PD group had the NoGo P3 approximately 69 ms late at Cz for the 2nd session (2nd: 545 ± 87 ms vs. 1st: 476 ± 128 ms, *p* = 0.04).

**Table 2:** Peak latencies of the NoGo N2 and P3 for the 1st and 2nd session in each group

		N2		P3	
		1st	2nd	1st	2nd
HC					
Fz		335 (56)	348 (61)	519 (70)	514 (68)
FCz		339 (56)	340 (55)	489 (47)	506 (50)
Cz		353 (57)	339 (76)	521 (75)	517 (66)
PD					
Fz		343 (95)	328 (75)	500 (56)	511 (80)
FCz		348 (75)	333 (68)	505 (59)	522 (85)
Cz		310 (120)	324 (79)	**476 (128)**	**545 (87)**
ICD					
Fz		336 (99)	340 (101)	548 (77)	603 (91)
FCz		361 (69)	342 (64)	500 (72)	532 (83)
Cz		312 (69)	301 (74)	543 (96)	535 (98)

Only the PD and ICD groups underwent DA treatment right after the 1st session

Numbers in boldface show their contrast with statistical significance *p* < 0.05

**Fig. 5:**
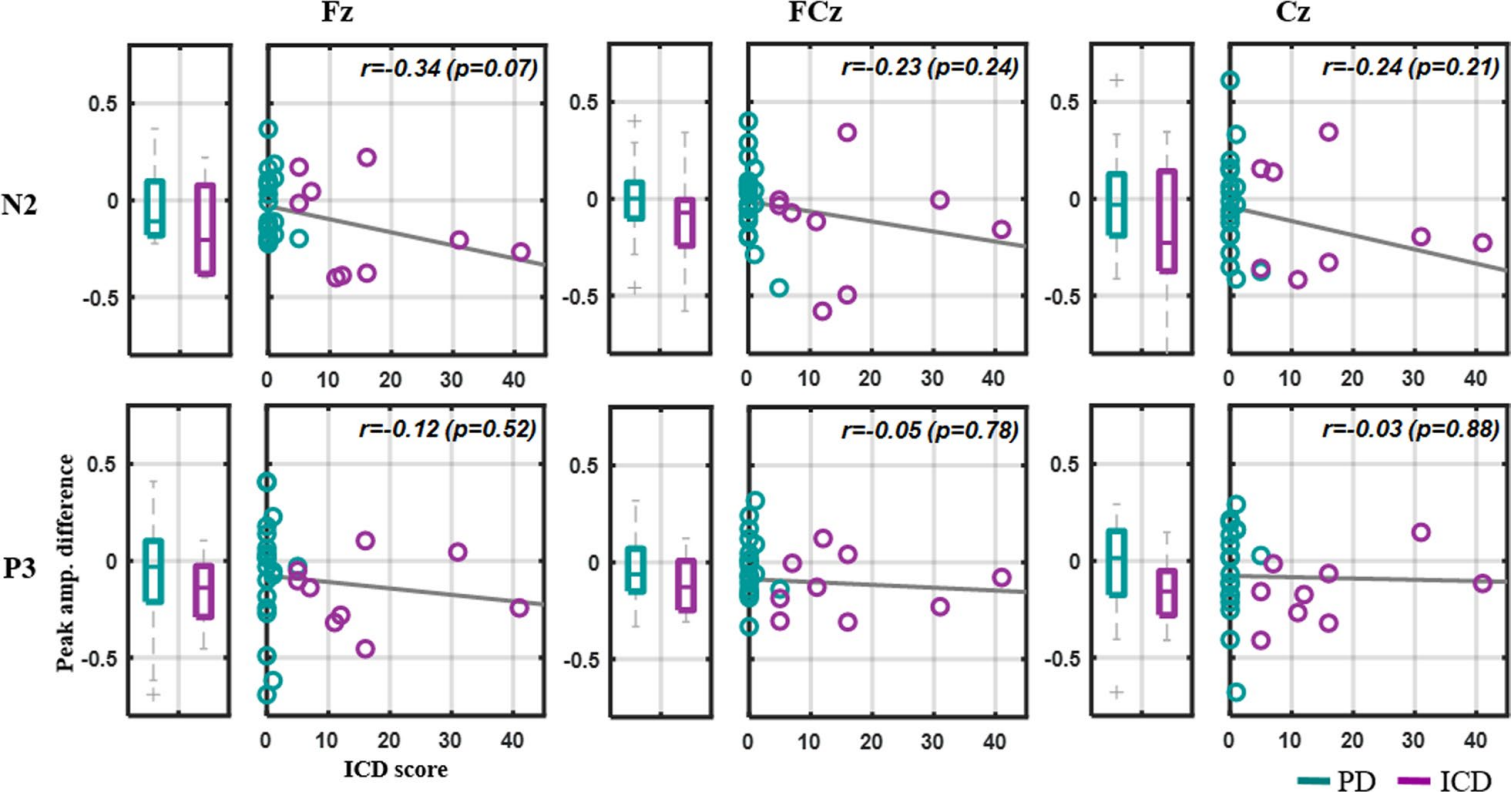
Between-session contrast of NoGo N2 and P3 peak amplitudes and their associations with ICD scores for the PD and ICD groups at the representative electrodes. Circles at the right side of each subplot represent the outcome for each PD and ICD individual (PD: 20, ICD: 9), whereas gray lines depict the linear relationships between the peak differences and ICD scores assessed by linear regression analysis

Figure 5 shows the between-session contrast in N2 and P3 peak amplitudes and their ICD score associations with the PD and ICD groups. The ICD group showed more deterioration in both N2 and P3 peak amplitudes relative to the PD group, yet, with less statistical significance (N2: 0.14 < *p* < 0.18, P3: 0.22 < *p* < 0.80). Most importantly, the between-session N2 decline correlated with the ICD scores, that is, the participants with a higher ICD score had a diminished signature after the DA treatment. Among the fronto-central electrodes, the extent of the N2 decline exhibited a marginal statistical association at Fz (*r* =  − 0.34, *p* = 0.07). This association barely emerged in the P3 counterpart.

## Discussion

This work contributed to the exploration of ERP-related features that can be used to reflect DA-triggered cognitive disorders in patients with PD. Furthermore, the non-medical grade lab-customized, cost-efficient LEGO-like EEG headset [29, 30] was successfully employed in this feasibility study. We found that patients with PD and ICD comorbidity exhibited N2 and P3 peak amplitude deterioration upon DA administration. The ICD severity tended to modulate the N2 deterioration. Therefore, these ERP findings objectively assessed the ICD adverse effect, which potentially constitutes a complimentary assessment to conventional scales, such as clinical interviews and self-reported questionnaires performed to patients with PD. The EEG wearability also facilitates neuro-monitoring in the living environments of patients with PD and facilitates the elaboration of an optimal pharmacological plan while chronically treating motor symptoms. The following paragraphs discuss the integrity of the ERP outcomes and feasible refinement towards the aforementioned purpose.

The stereotypical Go/NoGo protocol was employed to arouse both cognitive and motoric inhibition and to elicit the corresponding ERP signatures of N2 and P3 peaks at the fronto-central region, which manifested as a response to NoGo events (i.e., successful inhibitions) [13, 25, 26]. Accordingly, we implemented the two-target visual task. The NoGo N2 (200–400 ms) and P3 (400–600 ms) components noticeably appeared (*c.f.,* Fig. 3), in line with the literature. Furthermore, our study results showed that the PD groups (with and without ICD) exhibited weakened N2 and P3 peak amplitudes compared with the HC group (*c.f.*, Fig. 4), which replicated early findings [14, 32]. While previous studies mostly focused on EEG differences, either between different PD stages [33–35] or PD vs. HC individuals [14, 31, 32], fewer studies assessed typical patients with PD against those with ICD comorbidity [36]. To address this issue, we conducted two sessions of the Go/NoGo task interleaved with the DA-pharmacological treatment for PD groups. Contrastingly, only the ICD group tended a between-session decline in peak amplitude at the fronto-central midline electrodes (i.e., Fz, FCz, and Cz), particularly for the P3 counterpart, yet, with marginal statistical significance (*c.f.*, Fig. 4). Beyond the between-group comparison, the N2 peak deterioration was somehow modulated by ICD severity (i.e., patients’ self-reported QUIP-RS scores), which was statistically remarkable at Fz and marginally at Cz. The above comparison of PD versus PD and ICD comorbidity implied that the DA therapy made patients with ICD vulnerable to impulse control deterioration, evident both as behavioral manifestations (i.e., relatively faster yet mistaken responses in the 2nd session) and weakened fronto-central N2 and P3 peaks. Furthermore, we empirically demonstrated the potential of the cost-efficient EEG-sensing LEGO headset and of the ERP protocol and analytical framework for monitoring the impulse control capability of PD patients during the pharmacological intervention. It is noteworthy that the LEGO-like electrode-holder infrastructure [30] allows the unlimited reassemble of a compact EEG headset with minimal, yet, informative electrodes (Fz, FCz, and Cz only) on the scalp, thus removing redundant/irrelevant electrodes from other brain regions, improving headset wearability and comfort and offering individual optimization for each patient with PD if necessary.

Even though the above outcomes are encouraging, some issues should be mentioned and considered for future efforts toward the improvement of the effectiveness of the adopted protocol and framework. First, ERP waveforms are time-locked and phase-locked weak electrical potentials to stimulation events. By applying the synchronizing averaging to repetitively collected trials of the same task, the ERP components of interest (e.g., N2 and P3 signatures) can be revealed, since EEG background activity (i.e., concurrent to the engaged task) and/or accompanying random noises can be simultaneously greatly alleviated. Therefore, the number of collected trials and the extent of artifact contamination are two critical factors for the signatures’ signal-to-noise ratio (SNR). However, this work was a user-friendly EEG experiment for elderly participants (typically above 59 years). We reduced sessions to around 30 min and mounted the dry electrodes over the LEGO headset. As such, each single session only collected 72 NoGo trials per participant, which infrequently occurred (30%). The retained trials were even fewer after noisy trial removal (average removal: ~ 9%). Therefore, the limited trials inevitably downgraded the N2 and P3 SNR for certain participants. This potentially explains, in part, the noticeable intragroup and intergroup variability at the fronto-central electrodes (*c.f.*, Figs. 4B and 5). Future efforts should either incorporate advanced artifact removal or spatial filtering frameworks [47–50] for SNR improvement, given the number of EEG trials collected with this challenging recording setting. On the other hand, ERP signatures elicited by NoGo trials are highly driven by the engagement of attentional or working memory resources that are recruited by the Go/NoGo protocol configuration [51], namely, trial pace (i.e., stimulus-stimulus interval), the probability of NoGo trials, the difficulty of NoGo trials identification, and working memory load variability (i.e., varying the stimulus–response association), which reportedly affects the capability to elicit prepotent motor activity and probe inhibitory control [26, 51, 52]. Such factors are based on the assumption that the increase of the inhibitory effort to withhold responses to NoGo trials enhances cognitive inhibitory activity, following the model of reactive, selective inhibition of response [51, 52]. However, the psychological model of prepotent response inhibition is multifaceted, meaning that its underlying neural mechanisms may not be exclusively revealed in a standard Go/NoGo task [51, 52]. Exploring other inhibitory models is feasible by amending the experimental design; for instance, adding a pure block of Go trials only in the experimental design offers the capability to address the reactive, non-selective inhibition of response [36, 51, 52]. Future studies can capitalize both on the manipulation and optimization of the above designs, further diversifying and amplifying the between-session N2 and P3 contrast. Moreover, in addition to the time-related exploited ERP signatures, their inherent spectral oscillations could be worthy of research [36, 51]. For instance, the event-related spectral perturbations (ERSP) measure the time course of relative changes in the spontaneous EEG spectrum in response to stimulus events [53], i.e., ERP’s spectral energy. If ongoing EEG and ERP are not completely independent and/or stationary [53], the full-spectrum ERSP analysis can reveal the phase-incoherent event-related brain dynamics that cannot be fully explained by ERP. Some empirical demonstrations have been reported exactly in Go/NoGo task [54, 55]. Lastly, in contrast to the single-channel ERP analysis, multi-channel scenarios that exploit tempo-spatio-spectral characteristics among a set of channels of interest could be more reliable (e.g., functional connectivity [36, 56], linear source analysis [36, 51, 57]). Such multivariate signal analysis has been demonstrated to improve the efficacy of extracting event-relevant patterns from non-stationary or artifact-contaminated EEG activity [58–60].

## Limitations

This work has some limitations. The existence of ICD-related ERP signatures was empirically demonstrated in a relatively small number of ICD participants. Its generalizability has to be tested in a larger population in the future. Moreover, this work performed a single-day EEG recording and analysis. That is, each recruited participant partook in the Go/NoGo protocol with and without DA treatment only once. However, intra-individual differences in task-related EEG activities may present ecologically on a daily basis [61–64]. Several behavioral and psychological states, such as attention, stress, anxiety, and/or sleep quality may contribute to the above EEG non-stationarity. Effectively alleviating non-stationarity is still an open challenge [65–67], and, therefore, the ERP-marker’s robustness has to be elucidated over repeated measurements interspaced in the chronic pharmacological plans. Lastly, we mainly addressed the ERP signatures’ feasibility to reflect the ICD adverse effect in patients with PD. While considering the integrity of serving as biomarkers, a machine-learning framework (e.g., shallow or deep learning) has to be leveraged in the future to evaluate its discrimination ability in ICD detection from typical patients with PD.

## Conclusion

This work empirically demonstrated that the customized low-cost LEGO-like EEG headset enabled the extraction of ERP waveforms for the objective assessment of ICD in patients with PD during DA treatment. The ERP evidence may provide complementary information to behavioral evaluation, which is conventionally used to diagnose the ICD adverse effect.

## Data Availability

The datasets generated and/or analyzed during the current study are available from the corresponding author on reasonable request.

## Abbreviations

CE: Commission error
DA: Dopamine agonist
EEG: Electroencephalogram
ERP: Event-related potential
HC: Healthy control
H&Y: Hoehn and Yahr staging scale
ICD: Impulse control disorder
ICA: Independent component analysis
PD: Parkinson’s disease
QUIP-RS: Questionnaire for impulsive-compulsive disorders in PD-rating scale
RT: Response time
SE: Standard error
S&E: Schwab and England activities of daily living scale
UPDRS: Unified Parkinson’s disease rating scale
